# Making Crowns

**Published:** 1896-05

**Authors:** C. F. Rodgers

**Affiliations:** Conneaut, Ohio.


ARTICLE VI.

### MAKING CROWNS.

BY DR. C. F. RODGERS, CONNEAUT, OHIO.

I think the Hollingsworth system of crown- and bridge-work the best so far placed before the dental paofession, though its cost is an objection.

I have a system somewhat similar that I believe has merit.

Place pieces of pasteboard around your die-plate, having them wide enough to extend an inch above the plate, this fill very carefully with plaster. When hard you have an impression of your die-plate. Then drill a one-eighth inch hole through the plaster in the center of each tooth, beveling the upper edge. Now, with a sharp chisel cut off each tooth. To make the surface smooth, go over it lightly with a piece of fine sandpaper.

Tie the plaster on the die-plate and directly under each hole is the cusp of a tooth. Now warm both plaster and plate so that it can barely be held in the hand, and heating Mellott's metal to the melting point, pour in each hole quickly till all are filled. When hard break up the plaster and you will have perfect cusps, with a cone-shaped piece of metal attached. Remove them from the plaster, one by one, and cut off the cone of metal and place each cusp in its respective place on the die-plate. When all are in place warm a piece of base plate wax and place over all; invert the whole and press rather hard against your bench and lift up die-plate, this will leave all the cusps adhering to the wax. Take one at a time and place on the die-plate, with a few strokes of a file the little quarter inch piece of metal is removed and the cusp is flush with the die-plate. You now have just such cusps as are furnished with the Hollingsworth system. Keep them all on the piece of wax.

When you have a crown to make proceed as with the Hollingsworth system.

That is, make your band to fit the root, and finish the edge that is to go under the gum nearly as you wish it be when finished. Heat the band and immediately force the prepared end in a piece of soft wood and contour the crown while still in the wood. This will preserve the shape at the neck. After contouring, try in the mouth and select one of your little cusps. Articulate properly by filling band with wax and sticking on cusps. Have the band a little longer than you think necessary and file off till the cusp articulates properly. When you have the proper cusp selected, you do not have to make a special die for that crown, simply observe the position of your cusp and swedge cap on the same on die-plate.

When extracting teeth save those having good cusps; if they are too sharp, grind to shape with carborundum wheel, and saw cusp off with bracket saw. When you have thirty or forty of these cusps, cut a piece of tin to fit in a pasteboard box and shellac it on one side. Before the varnish dries place your cusps on it in regular order. Now fill all the little ridges between the cusps in which the plaster might catch and break with wax; oil the whole slightly, and carefully pour plaster till there is at least an inch above the tin. When hard, remove the plaster from the box, pick out cusps; this gives you a model of a die-plate. Send this to any place where they make fine gray iron castings, and for about a dollar you can have a die plate equal to a six-dollar one.—*Items of Interest.*

